# The Role of Low Self-Control and Risky Lifestyles in Criminal Victimization: A Study of Adolescents in South Korea

**DOI:** 10.3390/ijerph191811500

**Published:** 2022-09-13

**Authors:** Hyounggon Kwak, Eun-Kee Kim

**Affiliations:** 1 Division of Public Affairs and Police Administration, Dongguk University-Wise, Gyeongju 38066, Korea; 2 Department of Police and Law, PaiChai University, Daejeon 35345, Korea

**Keywords:** low self-control, routine activities, risky lifestyles, criminal victimization

## Abstract

In this study, we examine the links between low self-control, risky lifestyles, and victimization. Specifically, we explore a full mediation model to test whether risky lifestyles (unstructured activity, association with deviant peers, and delinquency) account for the effect of low self-control on victimization. For the current study, we apply structural equation modeling (SEM) to data from 1057 South Korean adolescents. The results indicate that low self-control only has an indirect effect on victimization, primarily through unstructured time and one’s own delinquency. Risky lifestyles were found to fully mediate the influence of low self-control on victimization. The findings demonstrate the utility of the integration of self-control with routine activities and lifestyle theories as a robust framework through which to examine victimization. Specifically, the results suggest that individuals maintaining low levels of self-control self-select into contexts that are conducive to victimization, increasing their attractiveness and suitability as targets for victimization in the absence of capable guardians.

## 1. Introduction

Adolescence is conceived as a developmentally critical stage in successful transition of individuals to adulthood and is associated with cognitive and biological changes. Furthermore, adolescence is related to an escalated desire for autonomy and independence that coincides with decreased parental control and increased peer influence. Although this is not necessarily problematic, as such independence involves normative developmental tasks during adolescence, certain lifestyles or routine activities are riskier than others, exposing adolescents to risky and vulnerable situations that create opportunity for crime.

Crime is not randomly observed across space and time; certain places and times are considered high-risk. Situational and opportunity theories suggest that crime is tied to individuals’ social activities, their interpersonal interactions, and the structure of the physical and social environment around them [1,2,3]. These elements create patterns of the spatial and temporal risk of crime. Because victimization risks are not equally distributed across time and space, variability in lifestyles and routine activities may result in a differential risk of victimization by unequally exposing individuals to risky people in risky places at risky times in unguarded situations [1,4]. Opportunity perspectives, including routine activity and lifestyle-exposure theories, provide frameworks through which to understand variation in the risk of victimization [1,3,5].

Contemporaneous victimization research incorporates another major perspective emerging from general theories of crime, namely self-control theory, suggesting that low self-control affects individual lifestyle choices and behavior, thereby making individuals increasingly vulnerable to victimization [6]. Although the integration of low self-control with routine activities and lifestyle theories has proven useful in predicting a variety of forms of victimization [7], the extent to which low self-control affects victimization remains unclear due because individuals with low self-control tend to self-select into risky situations. In particular, low self-control is assumed to be indirectly linked to victimization, primarily operating through intervening mechanisms, such as involvement in unstructured routines and delinquent lifestyles [6,7]. However, extant literature has yielded inconsistent and mixed results. Some researchers report that risky lifestyles only partially mediate the effect of low self-control on victimization, whereas a handful of studies show full mediation. These mixed findings demonstrate the need for further research involving closer examination of the links between low self-control, risky lifestyles, and victimization risk.

In addition, a limited number of empirical studies have examined the links between low self-control, risky lifestyles, and victimization in the context of South Korea; however, existing studies have only captured part of the risky/delinquent lifestyle picture, namely the association between criminal compatriots and delinquent behavior [8,9]. Although these factors are clearly important determinants of victimization risks, such behavioral patterns do not represent routine daily activities or lifestyle choices (e.g., unstructured activities or evenings spent out for leisure) made by many adolescents. The failure to consider a complete set of lifestyle measures assumed to mediate the link between low self-control and victimization may result in potential biases that preclude the generalizability of these results [7,10,11]. That is, the omission of direct measures of lifestyles/activities, such as unstructured activity (as a common cause of crime-centric outcome), may inflate or exaggerate the effects of low self-control, resulting in varying levels of observed mediation (partial versus full) across studies.

Such an approach is associated with certain ambiguities at the conceptual level (e.g., association with deviant peers). Specifically, the use of risky/delinquent measures may trigger theoretical indeterminacy and may reflect fundamentally distinct theoretical mechanisms [4]. For example, although the measure of deviant peer association may capture the concept of exposure to potential offenders, it may also reflect and confound multiple constructs, such as social processes in social learning/differential association theories, beyond situational opportunity. As a result, it would be difficult to empirically distinguish what is actually being tested through theoretical mechanisms without theoretically related measures of lifestyles/routine activities (e.g., unstructured activities). Taken together, the lack of theoretically relevant measures of lifestyle/routine activity can represent an obstacle not only the understanding of victimization but the development and assessment of the integrated perspectives of self-control and lifestyle/situational theories [7,12].

In this study, we attempt to address these limitations and fill the gaps in the extant literature on victimization by capturing the full array of theoretically relevant lifestyle measures. This results in a rigorous test of a low self-control–risky lifestyles framework, in addition to contributing to the generalizability of the integrative perspectives, increasing clarity and broadening its international scope. Using structural equational modeling (SEM) with a sample of 1057 South Korean youth, we propose a full mediation model to test whether low self-control is indirectly linked to victimization through the indicators of risky lifestyle or situational opportunity (e.g., unstructured routines, deviant peer association, and delinquent activities).

### 1.1. Risky Lifestyles/Routine Activities and Victimization

A considerable amount of work in the field of victimology has employed various opportunity theories—including routine activity theory and the lifestyle-exposure approach. Routine activity theory (RAT) posits that risk of crime increases with the convergence in space and time of a motivated offender, a suitable target, and absence of capable guardians [3]. The theory primarily describes the necessary conditions for a crime to occur; absent any of these criteria, opportunity for crime is considerably reduced. Unlike RAT, the lifestyle-exposure approach explains differential risk of victimization as a function of variability in lifestyles, leading to individuals being differentially exposed to dangerous places, times, and people in association with which crime is most likely to occur [1]. That is, the theory emphasizes variation in an individual’s lifestyle and the nature of one’s activities outside the home [1,7,13]. For example, adolescents who frequently go out without adult supervision, especially at night, and associate with deviant friends or engage in delinquent acts are at an increased risk of victimization compared to those who do not practice such risky lifestyles or activities [7]. Both theories are based on the idea that certain lifestyle and routine activities are riskier than others and create opportunities for criminal victimization, bringing people into contact with likely offenders in the absence of capable guardians [14].

Consistent with this idea, the existing body of research has indicated that time spent in unstructured routines in the absence of authority figures contributes to a heightened risk of adolescent victimization [14,15]. For example, those who frequently spend time away from home, go out at night alone, and walk around on the street are at increased risk of victimization due to their increased probability of coming into contact with motivated offenders in the absence of responsible guardians [15,16,17,18,19,20]. Because authority figures function as guardians against victimization and informal social control, involvement in unstructured activity can be a primary source of vulnerability to victimization, putting youths at an escalated risk of victimization [21,22,23,24].

In addition, association with criminogenic peers can increase adolescent risk of victimization. Engaging with delinquent peer groups not only increases the frequency with which youths interact with motivated offenders but enhances their attractiveness or suitability as targets in the absence of capable guardians [1,20,24,25]. For instance, youth gang members are more likely to experience victimization than nonmembers, making them vulnerable to retaliation [26,27]. Furthermore, there is evidence that individuals are frequently victimized by fellow members of their own delinquent peer groups [28]. Previous studies have provided empirical support for the relationship between deviant peer association and victimization risk, demonstrating that deviant friends increased the likelihood of victimization among adolescents, whereas conventional friends reduced the odds of victimization risk [19,20,22,23,24,29,30].

Similarly, deviant or delinquent lifestyles can place adolescents at an increased risk of victimization. Explanations of the offender–victim overlap typically emphasize shared lifestyles and routine characteristics between potential offenders and victims that produce likely pools of offenders and likely pools of victims. Thus, involvement in delinquent activities may affect the likelihood that an individual will be in close proximity to likely offenders by bringing them into contact with would-be offenders in an unguarded situations. This exposure or proximity to offenders increases the risk of victimization [1,19,31]. In addition, engaging in delinquent behaviors may make offenders suitable and ideal targets with relative impunity because they are unlikely to be considered legitimate victims by law enforcement or other authorities [31,32]. Moreover, they are less likely to call the police because of fear of their own implication in delinquent activities [19,31]. Previous research has documented that involvement in delinquent activities is among the strongest predictor of victimization, even when controlling for low self-control and other theoretically important factors [15,21,22,23,31,33]. For example, Sampson and Lauritsen [19] revealed that adolescent involvement in minor deviance and violent offending was a contributor to victimization, controlling for other lifestyle measures (e.g., leisure activities). In this respect, Lauritsen and colleagues pointed out that a delinquent lifestyle is the only lifestyle factor relevant to understanding adolescent victimization, as delinquent activities overwhelm all other lifestyle–victimization associations [34]. This line of research suggests that offending should be included as a causal antecedent of victimization, given that delinquent behavior is a stable predictor of adolescent victimization [13,22,31,35].

### 1.2. Integration with Low Self-Control Theory

Opportunity theories of victimization underscore lifestyles and daily behavioral patterns of individuals; what people do, with whom they associate, and how they behave places them at more or less risk of criminal victimization [1]. Lifestyle or routine behavior is indicative of an individual’s rationality, as careful people may avoid many misfortunes, whereas careless individuals may suffer from unexpected outcomes, such as victimization [1,36]. However, the theories say little about why certain people are more likely to adopt risky lifestyles that may put them at increased risk of victimization. A promising development in the victimology literature is the coupling of lifestyle/routine activity approaches with low self-control to provide improved understanding of the individual and situational contexts associated with victimization risk [6,7,24].

Gottfredson and Hirschi’s general theory of crime (also known as self-control theory) is arguably one of the most prominent and tested theories of crime causation, suggesting that individuals with low self-control are predisposed toward a wide range of criminal and analogous behaviors [37,38]. A variety of empirical evidence has provided support for this notion, demonstrating the robust relationship between low self-control and delinquency across a diverse body of populations [38].

Individuals who lack self-control tend to be impulsive, insensitive, nonverbal, shortsighted, and quick to anger [37]. Those with low self-control are likely to pursue risky behaviors without considering the potential long-term consequences of their actions. Schreck [6] outlined how the elements of low self-control influence the risk of victimization and contended that vulnerability to victimization is a byproduct of lifestyles and behavioral manifestations marked by low self-control. In other words, low self-control affects one’s lifestyle choices and the ways in which people behave, placing them in risky social settings and dangerous situations that may ultimately lead to the risk of victimization.

Time spent on unstructured activities can reflect an individual’s preferences or “here and now” attributes with respect to opportunity [2,6,39,40]. For example, activities requiring effort, skill, discipline, cognitive or mental exertion, and willingness to defer gratification are unlikely to be a top priority for people with low self-control [37]. Instead, high-risk opportunities that promise immediate gratification and rewards with little work and skill are viewed more favorably by those with low self-control [37,38]. As a result, those with low self-control are inclined to dislike settings that require discipline, supervision, or other constraints on their behavior; instead, individuals maintaining low levels of self-control favor unstructured/unsupervised time and gravitate toward delinquent peer groups and risky activities [7,24,39].

In terms of association with deviant peers, low self-control produces a range of negative social consequences in social institutions and personal relationships. For instance, those with low self-control tend to have difficulty making and keeping conventional friends and are therefore less able to sustain investment in conventional pursuits [37]. Due to the behavioral characteristics of low self-control (e.g., selfish, thoughtless, unreliable, and belligerent), adolescents who lack self-control are likely to be rejected by their peers and have few conventional peer choices, leading them to associate with others involved in delinquent acts [41,42]. Previous studies have provided evidence that people with low self-control tend to invest less time and energy in conventional pursuits and that they tend to have more deviant social networks and associate with individuals involved in deviant conduct [7,43]. Using panel data from the Gang Resistance Education and Training evaluation study (GREAT), Schreck and colleagues found that adolescents who possessed low self-control tended to have friendship with delinquents and engage in delinquent behavior, thereby escalating the likelihood of victimization [33].

Researchers have long noted the nexus between offending and victimization, as well as their shared predictive individual characteristics [39,44]. Recognizing the overlap between offenders and victims, scholars suggests that those with low self-control are not only more likely to expose themselves to risky situations that lack guardianship but also to engage in provocative behavior that may put them at heightened risk of victimization [6,33,45]. There is evidence indicating that those who are low in self-control tend to provoke antagonism and behave in ways that make them a more vulnerable target [46,47,48,49]. For example, Reisig and Pratt [50] discovered that individuals who are impulsive and insensitive to others were more likely to use profanity or offensive language in public places, which may enhance their risk of victimization by provoking interpersonal responses [45,51]. Similarly, a recent study by Felson and colleagues [45] illustrated that individuals with low self-control were more likely to be criminal victims because they were more frequently involved in verbal or interpersonal disputes and behaved in ways that provoked anger in others [47]. Taken together, the literature provides evidence that low self-control may translate into a heighted likelihood of engaging in certain lifestyles and risky behaviors that make people more vulnerable to becoming a victim of crime.

### 1.3. Nature of Effects and Generalizability

Since Schreck’s reformulation of self-control theory into a theory of vulnerability, a number of studies have demonstrated that low self-control is an important antecedent of multiple forms of property and violent victimization, providing a more complete picture of victimization. However, an important insight derived from the integrated perspective is that the effect of low self-control should be indirect, primarily through intervening variables associated with lifestyle and risky behavioral patterns [6,7], although extant literature yields inconsistent and mixed results. Some research has indicated that low self-control has a direct relationship with increased risk of victimization [25,52,53,54,55]. Most previous research on victimization, especially that conducted in the context of South Korea, has reported that low self-control has a direct effect on victimization and risky lifestyles, only partially mediating the effect of low self-control on victimization [8,9]. However, these previous studies have predominantly relied on narrowly defined measures of risky/delinquent lifestyles, namely peer deviance and one’s own delinquency. In contrast, other studies have uncovered a nonsignificant link between low self-control and victimization [11,23,39,56,57]. This line of work suggests that low self-control only indirectly affects victimization risk through intervening variables.

The mixed findings of low self-control effects observed in previous victimization literature is perhaps due to failure to capture the full array of relevant lifestyle or behavioral patterns. A recent meta-analysis conducted by Pratt and colleagues [7] concluded that the effect of low self-control on victimization should be indirect when including direct measures of intervening causal mechanisms that link low self-control and victimization. Supporting a full mediation hypothesis, Turanovic and Pratt [39] indicated that the predictive effects of low self-control on violent victimization became nonsignificant when accounting for each of the risky lifestyles (e.g., unstructured activities, deviant friends, and delinquent behavior), suggesting that risky lifestyles fully mediate the effects of low self-control on victimization. This finding is supported by Reisig and Golladay [11], who revealed that risky lifestyles (e.g., unstructured activities, peer deviance affiliation, and conflict escalation) fully accounted for the relationship between low self-control and victimization. Similarly, Fox and Bouffard [23] discovered that low self-control influenced victimization indirectly by facilitating adolescents’ unstructured/unsupervised time, exposure to deviant friends, and involvement in deviance. Prior research is generally supportive of hypotheses derived from low self-control/lifestyle theory, although the degree to which the relationship between low self-control and victimization is mediated by risky routines remains relatively unexplored in the Korean context. Given the continued influence of integrative perspectives, further research is clearly warranted.

### 1.4. The Present Study

**Figure 1:**
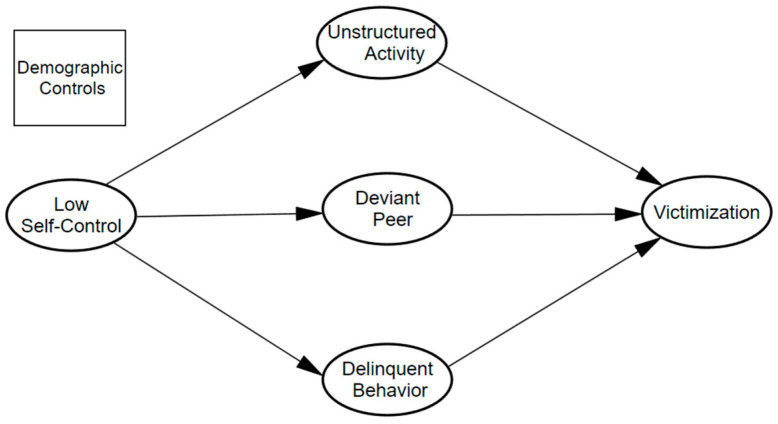
Conceptual model for the effects of low self-control and lifestyle/routine activities on victimization.

In the current study, we explore a full mediation model to test whether low self-control indirectly affects victimization through risky lifestyles (unstructured activity, association with deviant peers, and delinquent behavior), controlling for other demographic factors. The conceptual model guiding the current research is presented in Figure 1. Unlike previous research, in this study, we frame the indirect effect of low self-control on victimization as originally implied by Schreck [6] and investigate whether the mediation (indirect) hypothesis is the best fitting model. An alternative model is also tested in which direct effects of low self-control on victimization are included.

## 2. Materials and Methods

### 2.1. Participants

Data examined in the current study came from the Study on Child Delinquency, a study of a school-based sample of children in grades 4 through 6 attending 15 schools in Seoul, South Korea. This self-report survey was administered by the Korean Institute of Criminology (KIC) in 2009. To gather a representative sample within the city, cluster sampling methods were utilized. The city was divided into 5 districts according to income levels. These five districts contained 15 boroughs among the 25 boroughs in Seoul. A public elementary school was randomly selected from within each borough. Any school refusing to participate in the survey was replaced with the next school included in the sampling frame. As a result, a total of 15 elementary schools were included. Within each selected school, one class was randomly selected from grades 4, 5, and 6, resulting in a total sample of 1128 students. For the current study, seventy-one cases were excluded from the analyses due to insufficient data on key variables of interest. A few respondents did not answer some items, resulting in a small amount of randomly missing data (less than 1.3% of cells in the dataset). The missing values were replaced by estimated values using the expectation–maximization (EM) approach [58,59], which resulted in a final sample of 1057 students for analyses. The sample was nearly evenly divided among boys (49.6%) and girls (50.4%). The age of the respondents ranged from 11 to 13 years, with an average age of 12 years. Approximately, 91% of respondents lived in intact families.

### 2.2. Measures

#### 2.2.1. Dependent Variable

The latent construct, i.e., victimization, was assessed with two indicator variables: personal victimization and property victimization. Personal victimization was measured with five items by asking respondents to indicate how many times during the preceding year they had experienced the following events (Cronbach’s α = 0.701): (1) being beaten up by someone, (2) being threatened, (3) being collectively bullied and intentionally excluded from a group, (4) being sexually harassed, and (5) having had something damaged or destroyed on purpose. Property victimization was measured by three items (Cronbach’s α = 0.650): (1) had money or things stolen from me, (2) had my pocket picked or money snatched, and (3) had money and things taken from me by a threat. Each item was answered on the ordinal response scale, ranging from 0 (never) to 4 (7 times or more). The responses to the items were summed to construct the measures of personal and property victimization. The natural log (plus a constant of 1) of property victimization indicator was taken to address nonnormality, and skewness was reduced from 4.97 to 2.23. Higher scores on the latent variable of victimization scale indicate that the respondents have experienced more victimization in general.

#### 2.2.2. Independent Variables

*Low self-control.* Low self-control was constructed as a 10-item composite measure that reflects a general personal trait for the current study. Consistent with previous research [6,60], ten items were included to construct the scale of low self-control, capturing various components of low self-control: impulsivity, volatile temper, self-centeredness, and preference of a simple task (Cronbach’s α = 0.818). These questions encompassed: (1) “I tend to do things first that bring me the pleasure now”; (2) “I tend to quit when things get laborious and complicated”; (3) “I tend to do whatever I want even when it is causing problems for other people”; (4) “I sometimes feel the impulse to hit or hurt someone”; (5) “I lose control over myself when I am angry”; (6) “I tend to get irritated or annoyed easily, even by little things”; (7) “I have difficulty remaining seated;” (8) “I tend to get restless and keep moving”; (9) “I have difficulty keeping attention on one thing”; and (10) “I have trouble getting work done”. These items were measured on a five-point Likert scale with responses ranging from 1 (strongly disagree) to 5 (strongly agree). The responses to these questions were summed, with higher scores representing lower levels of self-control.

*Unstructured activities.* Unstructured or unsupervised activities are defined as activities with no particular purpose or agenda with respect to how much time is to be spent [2,61]. Following the lead of previous research [21,30,36,39,61], unstructured activity was measured by combining three items that asked respondents how many times, on average, per week they were involved in the following activities after school/at night (Cronbach’s α = 0.692): (1) roam around on streets without a particular purpose, (2) loiter or hang around outside home for a long time, and (3) go out and move around from place to place until late at night. The responses for these items ranged from 0 (never) to 5 (more than 5 times). An index of unstructured activity was created by summing these three items, which was then transformed to its natural logarithm (plus a constant of 1) to address the nonlinearity between the independent variable and victimization. Higher scores indicate more time spent on unstructured activities.

*Deviant Peer Association.* The latent variable of deviant peer association was constructed with two manifest variables: peer delinquency and misbehavior. Respondents were asked to indicate the extent to which they agreed that their close friends engaged in delinquent activities (Cronbach’s α = 0.730): (1) “My close friends hit others quite often”; (2) “My close friends bully others quite often”; and (3) “My close friends smoke cigarettes and drink alcohol quite often”. A peer misbehavior measure was also created using three items (Cronbach’s α = 0.686): (1) “My close friends swear or use foul language quite often”; (2) “My close friends are scolded by teachers quite often”; and (3) “My close friends get into physical fights quite often”. Response categories for these questions ranged from 1 (strongly disagree) to 5 (strongly agree) and were summed to generate each observed variable, delinquent activities and misbehavior, which served as indicators of deviant peers, with higher scores representing more deviant peers [24].

*Delinquency.* The latent construct of deviant behavior was measured by three indicator variables, reflecting how many times respondents had engaged in three types of delinquent behaviors during the last 12 months: violent behavior, disruptive behavior, and conflict behavior. Violent behavior was captured with four items (Cronbach’s α = 0.665): (1) beaten up someone, (2) got into a physical fight with others, (3) involved in a group fight, and (4) sexually harassed someone. Disruptive behavior was constructed as a measured with four items (Cronbach’s α = 0.571), including (1) purposely damaged or destroyed property or things that did not belong to you, (2) intentionally damaged or destroyed property or things that belong to a school, (3) intentionally scratched others’ car or house, and (4) been loud, rowdy, or unruly in a public place. Conflict behavior was assessed by three items (Cronbach’s α = 0.668), including (1) had an argument or quarrel with others, (2) cursed at other people, and (3) bullied and picked on others [61]. Individual responses to these questions ranged from 0 (never) to 4 (more than 7 times). These items were summed to create each observed indicator, with higher values indicating greater involvement in delinquency. Violent and disruptive behaviors were transformed using logarithmic transformations (plus a constant of 1) to ensure the assumption of normality.

*Control Variables*. Several demographic factors were included as controls. Gender was treated as a binary variable, with male respondents coded as 1, whereas age was measured in years. Family structure was coded as 1 if the adolescent lived with two married parents and 0 if respondent lived in other family structures, such as with divorced or separate parents.

### 2.3. Analytic Strategy

Given the research purpose and the proposed hypotheses, in the current study, we utilized structural equation modeling (SEM) to test direct and indirect effects of low self-control on victimization through the indicators of risky lifestyles. SEM provides several benefits for analysis, such as allowing researchers to evaluate the hypothesized model by simultaneously testing for direct, indirect, and total effects of exogenous and endogenous variables [62]. In addition, it provides more reliable and accurate estimates of the relationships between primary constructs by taking unreliability and measurement error into account in the model [58]. Thus, the SEM is an optimal technique for the current study.

In the present study, several analyses were performed to examine the indirect effects of low self-control on victimization via risky lifestyles. First, a measurement model was developed and tested using confirmatory factor analysis to determine the adequacy of the underlying latent construct. Second, a structural equation model was estimated with maximum likelihood (ML) estimation to examine whether the main independent and mediating variables significantly influenced victimization risk. Finally, to evaluate the statistical significance of the indirect effects, mediation tests were conducted using bootstrapping methods with bias-corrected confidence estimates. The 95% CI (confidence interval) of the indirect effect was obtained with 2000 replications for bootstrap resamples [63,64].

Goodness of fit of the models was assessed using the $\chi^{2}$ test, the comparative fit index (CFI; good fit > 0.95, adequate fit > 0.90), the goodness-of-fit index (GFI; good fit > 0.95, adequate fit > 0.90), standardized root mean square residual (SRMR; good fit < 0.06), and root mean squared error of approximation (RMSEA; good fit < 0.06, adequate fit < 0.08) [62,65]. Chi-square difference tests for nested models were used to compare alternative models. In addition, each model was screened for Heywood cases (e.g., implausible values, such as negative variance estimates). According to findings reported in the literature, the structural model allowed for correlation of the disturbances of unstructured activity, deviant peers, and delinquent behavior.

## 3. Results

### 3.1. Measurement Model

A measurement model was tested with all the latent variables of the structural model [66]. The fit indices suggest that model fit the data well, with $\chi^{2}$ (19) = 78.291, *p* = 0.00, GFI = 0.984, CFI = 0.972, SRMR = 0.026, and RMSEA = 0.054; 90% CI = [0.042, 0.067]. Although the $\chi^{2}$ value is statistically significant and suggests a lack of model fit, it is highly affected by sample size and might be a reflection of its sensitivity to a large sample size [62]. In this case, the critical N statistic should be used as an alternative indicator, and a model with a critical N of 200 or more represents an acceptably adequate model fit [67]. The CN value for the measurement model was 489, suggesting an overall good fit of the model to the data.

**Figure 2:**
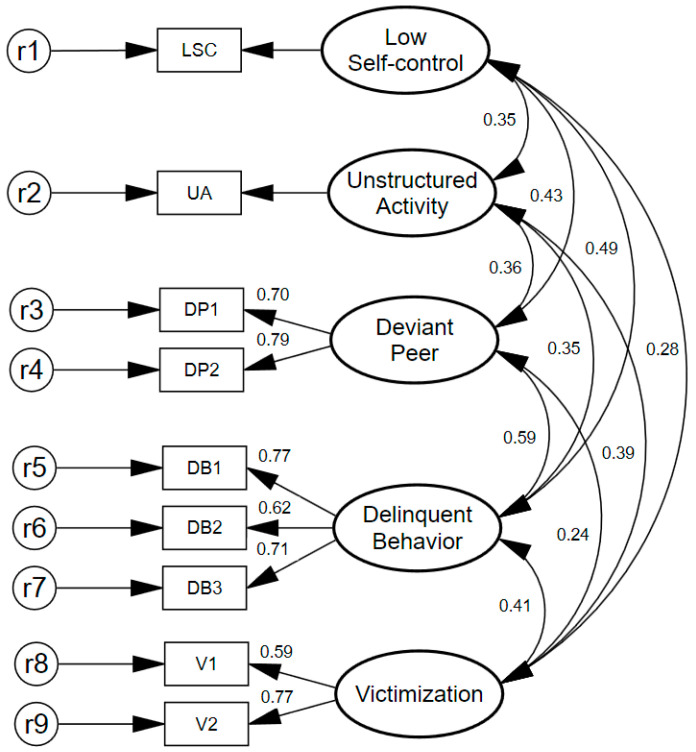
Measurement model for total sample with standardized coefficients (N = 1057). Circles represent latent constructs, and rectangles represent observed variables. $\chi^{2}$ (19) = 78.291, *p* = 0.00, GFI = 0.984, CFI = 0.972, SRMR = 0.026, CN = 489, AIC = 130.291, and RMSEA = 0.054; 90% CI = [0.042, 0.067].

Figure 2 presents the results of the measurement model with correlation coefficients between the latent factors and factor loadings for the construct indicators. All factor loadings for latent variables were above 0.500 and statistically significant (*p* < 0.001), indicating that all of the selected indicators are acceptable in terms of the extent to which they appropriately reflect their underlying concept. There were no Heywood cases, and the indicators for low self-control and unstructured activity were set to 1.00.

It is important to note that the correlation between low self-control and involvement in delinquency (r = 0.485, *p* < 0.001) is stronger than the correlation between low self-control and victimization (r = 0.278, *p* < 0.001). Furthermore, association with deviant peers is moderately correlated with victimization (r = 0.243, *p* < 0.001) and strongly correlated with delinquent involvement (r = 0.587, *p* < 0.001). Additionally, the correlation between delinquent behavior and victimization is strongest among the indicators of risky lifestyle. Given the adequate relationships between the observed indicators and the underlying latent constructs in the measurement model, the structural relationships are estimated in the next section.

### 3.2. Model Testing: Conceptual Model

**Figure 3:**
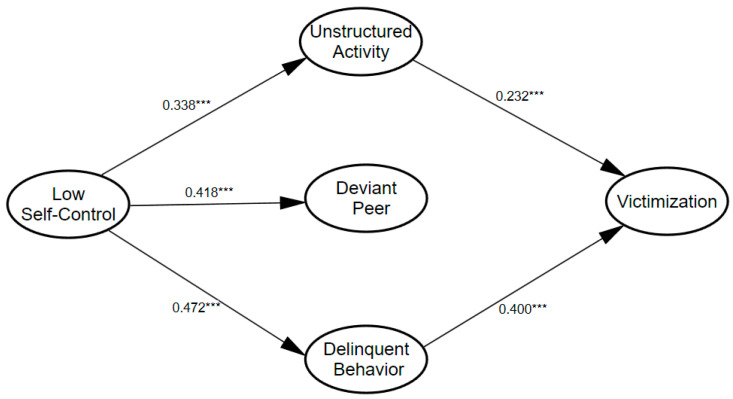
Structural model (N = 1057). Note: $\chi^{2}$ (32) = 140.957, *p* = 0.00, GFI = 0.978, CFI = 0.953, SRMR = 0.029, CN = 347, and RMSEA = 0.057; 90% CI = [0.047, 0.067]. Values are presented as standardized coefficients, and only statistically significant paths are shown. All other covariates are controlled but not presented in the model. *** *p* < 0.001.

A structural model was estimated to test the applicability of the integrative framework to a South Korean sample. As illustrated in Figure 1, the hypothesized conceptual model was estimated to examine whether the effect of low self-control on victimization was fully mediated by intervening mechanisms, controlling for all relevant covariates (demographic controls). An alternative model was also created to test whether low self-control really had only an indirect effect on victimization. In the alternative model, a direct path from low self-control to victimization, was added and estimated. The fit statistics for the initial/conceptual model indicate that the model fits the data well, with $\chi^{2}$ (32) = 140.957, *p* = 0.00, GFI = 0.978, CFI = 0.953, SRMR = 0.029, and RMSEA = 0.057; 90% CI = [0.047, 0.067]. The alternative model, which included a parameter linking low self-control to victimization, also indicated that the overall model fit the data well: $\chi^{2}$ (31) = 139.850, *p* = 0.00, GFI = 0.978, CFI = 0.953, SRMR = 0.030, and RMSEA = 0.058; 90% CI = [0.048, 0.068]. The two models had a nearly equivalent fit. However, in the alternative model, the direct path from low self-control to victimization was not statistically significant (t value was 1.079). In addition, the $\chi^{2}$ difference between these two models was not statistically significant ($\Delta \chi^{2}$ = 1.107, df = 1), which indicates that the hypothesized initial/conceptual model was more parsimonious, favoring the initial/conceptual assumption of a fully mediated model rather than the alternative model. The results of the hypothesized model are presented in Figure 3 with the standardized coefficients.

As previously noted, low self-control did not directly affect the level of victimization; however, low self-control had a statistically significant indirect influence on victimization though engagement in unstructured routines and delinquent activities (indirect bootstrapped β = 0.242, *p* < 0.05, 95% CI = 0.172, 0.308). More specifically, low self-control significantly predicted all the three indicators of risky lifestyles. Low self-control increased time spent on unstructured routines (β = 0.338, *p* < 0.001), association with deviant peers (β = 0.418, *p* < 0.001), and involvement in delinquency behaviors (β = 0.472, *p* < 0.001), even after controlling for other covariates. In addition, two indicators of risky lifestyles had direct and positive effects on victimization; unstructured activity (β = 0.232, *p* < 0.001) and delinquent behavior (β = 0.400, *p* < 0.001) were significantly associated with heightened risk of adolescent victimization. In contrast, the effect of deviant peer association on victimization was not significant. In terms of control variables, age (β = −0.188, *p* < 0.001) and sex (β = 0.083, *p* < 0.05) were significantly associated with victimization risk.

## 4. Discussion

Numerous empirical studies have verified that an individual’s level of self-control and risky lifestyles are important correlates of victimization risk [7], although the nature of this relationship has not been entirely explained. The main purpose of this study was to examine whether the integrated model linking low self-control, risky lifestyles, and victimization is generalizable to other social and cultural contexts, especially in South Korea. Overall, the results of the current study contribute a layer of support for the utility of the integration of self-control with lifestyle/routine activities perspectives as a robust framework through which to examine individual difference in the risk of victimization.

Although prior studies conducted in the context of South Korea have suggested a direct relationship between low self-control and victimization [8,9], the results of the current study demonstrate that low self-control had no direct effect on victimization. Rather than a direct effect, the findings reveal that low self-control only had an indirect effect on victimization, primarily through two intervening mechanisms: involvement in unstructured activities and delinquent behaviors. This finding is in line with integrative perspectives, suggesting that the risk of victimization is a byproduct of low self-control, as it affects individuals’ lifestyle choices and behaviors, resulting in vulnerability to victimization [6,7]. More specifically, low self-control was associated with risky lifestyles. Youths with low self-control tended to allocate their time to unstructured/unsupervised activities with deviant peers away from responsible authority figures and engage in criminal behavior. These risky activities, particularly unstructured activity and delinquent lifestyles, lead increased risk of adolescent victimization. These findings highlight that risky lifestyles/routine activities, such as unstructured activities, association with deviant peers, and delinquency, leading to victimization might reflect an individual’s preference and personality, as such activities may provide pleasurable short-term benefits and immediate gratification [2,6,37].

In addition, the results reveal that involvement in delinquent behaviors is the most victimogenic of all risky activities. This finding seems to support the idea that the overlap or interrelationships between offending and victimization may be the result of shared risk factors, namely low self-control [34,44,45]. The shared overlap may be also due to increased opportunities (e.g., interaction with motivated offenders). Once in risky social settings, those with a deficit in self-control might behave in ways that provoke grievances or disputes, which may make them more attractive and suitable targets, escalating their susceptibility to victimization [6,39,45,46]. It is also conceivable that those who are involved in deviant activities may be perceived as suitable targets because of relatively minimal fear that they are likely to mobilize the legal system [31,32]. In other words, risky social settings imply that delinquent youth are exposed to victimization risks not only because of their propensity for active victim precipitation but also because of the exposure itself. Thus, it is not only when, where, and with whom adolescents spend their time that explains their victimization but also how they behave toward others [11,45,51,68,69].

Involvement in crime as an offender or victim tends to peak in teenage years [31,34]. However, it is important to note that crime peaks in adolescence do not necessarily imply that low self-control peaks at this time; instead, what might peak is the opportunity for crime, perhaps because of increased independence from parents [19,31,34]. In this sense, victimization is an undesirable outcome and a qualitative distinct phenomenon from offending, in that it is not in anyone’s self-interest to be a criminal victim [6,7]. Thus, having low self-control could translate into certain risky lifestyles and behaviors that make an individual more vulnerable or a suitable target because deficit in self-control may preclude an awareness of potential deleterious outcomes of their own risky behaviors.

Taken together, the results of the current study confirm the logical compatibility of self-control theory with lifestyle/routine or opportunity approaches in accounting for variation in the risk of adolescent victimization. The indirect effect of low self-control suggests that the measures of lifestyle or situational opportunity are more proximate causes of and have stronger effects on victimization risks among youths than do the more distant causes, including low self-control [7,11,52,56]. Thus, future research on victimization should include low self-control measure as a common cause of lifestyle and victimization. Simply put, individual and situational factors should not be considered in isolation in victimization research, as low self-control indirectly increases the risk of victimization by influencing various aspects of an individual’s lifestyle and behavioral choices [6,7,39].

In addition, the indirect relationship between low self-control and victimization suggests that earlier models applied in the context of South Korea may have been underspecified; with the use of complete risky lifestyle measures, including unstructured routines and conflict escalation behavior, conclusions about low self-control may differ. Thus, future research investigating the link between low self-control, risky routines, and victimization should include a broad array of behavioral routines to avoid mis-specification in modeling differential risks of victimization among youths.

Crime victimization among adolescents remains a significant issue in South Korea, and youth are among the most criminally victimized segment of the population compared to other age categories [34,48,70]. Given the research findings, it is clear that efforts to reduce victimization risks should focus more on changing problematic routine activities rather than changing individual levels of self-control. This might be a much more feasible and practical solution to preventing victimization among youth. On the other hand, exposure to risk is not simply a byproduct of risky lifestyles or routine activities, given that these risky daily routines are, to some extent, a matter of choice, influenced by individual traits or propensities. Furthermore, individuals with low self-control are less likely to change their risky lifestyle behaviors that may reduce the risk of victimization [33]. As such, any successful crime prevention strategy should build on an integrated approach with a dual focus. Despite the relative stability of self-control, scholars suggest that personal traits can be modified [71]. In this regard, effective prevention and intervention efforts aimed at improving self-control seem to be equally critical with respect to reducing victimization risk, given that individual propensity and exposure are developmentally related.

Despite the contributions of this study to the growing body of literature concerning the integration of self-control with lifestyles/routine activity approaches, the current study is subject to several limitations that merit further discussion. First, an important caveat related to the use of cross-sectional data is that the temporal order between the variables is not established. Namely, the victimization items asked about the preceding year, whereas low self-control and two of the lifestyle/routine activity measures (unstructured activities and deviant peer association) were measured at the time of the survey. Thus, longitudinal studies are warranted to elaborate causal models, which could yield important insights into the temporal relationships between low self-control, risky lifestyles, and victimization. Furthermore, the models only capture a short snapshot of the inter-relationships between low self-control, risky lifestyles, and victimization risk during adolescence (ages 11 to 13). Whether the pattern of findings obtained in the current study continue into early and middle adulthood remains an empirical question. Thus, it is important to interpret these findings with such issues in mind.

Second, although the measure of deviant behavior entails provocative and conflict escalation behaviors that immediately precede victimization, it is unclear how individuals with low self-control act once they self-select into risky situation, and it is also unclear whether youths were actually victimized in the context of a delinquent behavior. It is possible that low self-control and indicators of risky lifestyles may interact such that individuals with low self-control might be more likely to respond to criminogenic and situational cues with offenders. Thus, additional research is needed to examine how individuals with low self-control actually behave when exposed to risky settings.

Finally, future research should explore other potential personality traits or psychological characteristics, such as anxiety, depression, neuroticism, agreeableness, conscientiousness, or honesty–humility, which have been found to be associated with lifestyles and criminal events as both offenders and as victims [72,73]. These traits, in addition to low self-control, may help to further explain what makes individuals vulnerable to crime.

## 5. Conclusions

Despite these limitations, this study contributes to the literature in two substantive ways. First, the present study increases the generalizability of the integrated framework through its application to a sample of South Korean adolescents. This application allowed for testing of the utility of the framework with respect to understanding general victimization. Second, the current study explores the extent to which the influence of low self-control on victimization is mediated by risky lifestyles. Overall, this study provides support for an integrative/holistic approach that combines explanations of individual trait and situational risk factors into a unified dynamic model. As found in the current study, individual traits and personality factors are relevant with respect to victimization, although only to the extent that they influence various social and behavioral processes. In other words, individuals are not randomly placed in risky or vulnerable social settings; instead, they seem to select contexts consistent with their own preferences and personality. Victimization is recognized as a psychologically harmful, physically damaging, and socially isolating aspect of the life of adolescents [74,75]. Despite these adverse consequences of victimization, young adolescents are the least likely group to report their victimization to police and others, making it more difficult to prevent its adverse impact in earlier stages [68]. Thus, it is essential for scholars to completely understand the etiology of adolescent victimization and life processes that place them at heightened risk of being victimized [76,77,78]. Identifying risk factors related to victimization with young adolescents can promote adolescents’ successful transition to healthy adulthood by providing practical preventive measures.

## Data Availability

Not applicable.
